# *Lactobacillus plantarum* surface-displayed ASFV (p54) with porcine IL-21 generally stimulates protective immune responses in mice

**DOI:** 10.1186/s13568-021-01275-9

**Published:** 2021-08-12

**Authors:** Xiao-Lei Chen, Jun-Hong Wang, Wei Zhao, Chun-Wei Shi, Kai-Dian Yang, Tian-Ming Niu, Gui-Lian Yang, Xin Cao, Yan-Long Jiang, Jian-Zhong Wang, Hai-Bin Huang, Yan Zeng, Nan Wang, Wen-Tao Yang, Chun-Feng Wang

**Affiliations:** College of Veterinary Medicine, College of Animal Science and Technology, Jilin Provincial Engineering Research Center of Animal Probiotics, Key Laboratory of Animal Production and Product Quality Safety of the Ministry of Education, Jilin Agricultural University, 2888 Xincheng Street, Changchun, 130118 China

**Keywords:** ASFV, p54, Recombinant, *L. plantarum*, Immune evaluation

## Abstract

African classical swine fever virus (ASFV) has spread seriously around the world and has dealt with a heavy blow to the pig breeding industry due to the lack of vaccines. In this study, we produced recombinant *Lactobacillus plantarum* (*L. plantarum*) expressing an ASFV p54 and porcine IL-21 (pIL-21) fusion protein and evaluated the immune effect of NC8-pSIP409-pgsA'-p54-pIL-21 in a mouse model. First, we verified that the ASFV p54 protein and p54-pIL-21 fusion protein were anchored on the surface of *L. plantarum* NC8 by flow cytometry, immunofluorescence and Western blotting. Then, the results were verified by flow cytometry, ELISA and MTT assays. Mouse-specific humoral immunity and mucosal and T cell-mediated immune responses were induced by recombinant *L. plantarum*. The results of feeding mice recombinant *L. plantarum* showed that the levels of serum IgG and mucosal secreted IgA (SIgA), the number of CD4 and CD8 T cells, and the expression of IFN-γ in CD4 and CD8 T cells increased significantly, and lymphocyte proliferation occurred under stimulation with the ASFV p54 protein. Our data lay a foundation for the development of oral vaccines against ASFV in the future.

**Supplementary Information:**

The online version contains supplementary material available at 10.1186/s13568-021-01275-9.

## Introduction

African swine fever (ASF) is a highly infectious disease that has caused great economic losses to the animal husbandry economy. Since it was first discovered in Kenya in 1921, 68 countries have been affected by ASF. ASF entered China on August 3, 2018, causing great economic losses to China's animal husbandry (Wang et al. 2018a, b; Zhao et al. 2019). ASFV is the only member of the genus *Asfivirus* in the *Asfarviridae* family and spreads between *Ornithodoros moubata* ticks and warthogs (Bonnet et al. 2020). ASFV infects wild and domestic pigs of all breeds and ages. The clinical manifestations of diseased pigs are fever, skin cyanosis, and obvious bleeding in lymph nodes, kidney, and gastrointestinal mucosa, and the mortality rate is as high as 100% (Muangkram et al. 2015).

The ASFV genome encodes 150 to 200 proteins, approximately 50 of which are structural proteins. P54 plays an important role in attachment, entry and replication (Jia et al. 2017). Some studies have shown that the p54 protein located in the inner envelope of the virus has good immunogenicity (Petrovan et al. 2020). Some researchers have used the p54 protein and p30 protein to construct recombinant subunit vaccines to protect some immunized pigs from the lethal challenge of ASFV (Argilaguet et al. 2012). Recent studies have shown that the construction of recombinant *Saccharomyces cerevisiae* (*S. cerevisiae*) expressing the p54 protein can induce mucosal immunity and produce strong antibodies in pigs (Chen et al. 2020). However, due to the complexity of the ASFV virus, the existence of multiple genotypes and the limited effect on protective immunity, no ASFV vaccine is available (Rock. 2017; Sang et al. 2020). The findings of researchers in 2019 provide a comprehensive molecular model for the capsid structure of ASFV. It is hoped that this information will contribute to the development of future strategies, including vaccines (Liu et al. 2019).

Interleukin 21 (IL21) is a cytokine mainly produced by differentiated mature CD4^+^ T cells and natural killer T cells (Battaglia et al. 2014; Wang et al. 2015). Some studies have found that IL-21 can enhance the expression of CD86 in mouse B cells so that it can provide superior T cell costimulatory ability (Attridge et al. 2014; Tangye and Ma 2020). There is evidence that IL21 participates in B cell activation and proliferation through its specific receptor (IL21R) and regulates germinal center and humoral immunity (Tangye et al. 2017), while lack of IL-21 leads to memory B cell deficiency (Erman et al. 2015; Moens and Tangye 2014). In summary, IL-21 is a potential vaccine adjuvant.

Lactic acid bacteria are the most commonly used probiotics, which can maintain intestinal homeostasis and enhance immunity (Vinusha et al. 2018). Compared with traditional attenuated vaccines, recombinant *L. plantarum* based on probiotics is considered to be an excellent tool for veterinary vaccines (Yang et al. 2017), which can induce a strong immune response by surface anchored expression of antigen, while *L. plantarum* NC8 isolated from silage has a high ability to express foreign protein (Yang et al. 2017). To construct recombinant *L. plantarum* that can express foreign antigens, our laboratory used the truncated anchor sequence of poly-γ-glutamate synthase A' (PGSA') (Cai et al. 2017), which is a transmembrane protein from *Bacillus subtilis* and has high display efficiency (Lei et al. 2015). For instance (Jiang et al. 2017), *L. plantarum* has been used to express conserved M2E and HA2 fusion antigens to induce protection against influenza viruses (Yang et al. 2018a, b, c). Expression of the S-DCpep fusion protein against transmissible gastroenteritis virus (Yang et al. 2018a, b, c) expressing the porcine epidemic diarrhea virus S gene can improve the immune response of the body (Jin et al. 2018). In this study, a recombinant *L. plantarum* strain that can express the ASFV p54 protein and p54-pIL-21 fusion protein was designed to evaluate the immune effect of mice in order to lay a theoretical foundation for the development of oral vaccines.

## Materials and methods

### Animals and ethical statement

The animals used in this experiment were purchased from HFK Bioscience Co., Ltd. (Beijing, China). Pathogen-free female C57BL/6 mice aged 6 to 8 weeks were raised in SPF rooms. The whole animal experiment met the requirements of the Animal Management and Ethics Committee of Jilin Agricultural University.

### Construction of recombinant *L. plantarum*

To construct recombinant *L. plantarum*, the gene sequences of porcine interleukin IL-21 (pIL-21) (gene number: AB073020.1) and p54 (gene number: 22220355) of African classical swine fever virus were synthesized and purified by GENEWIZ (Jiangsu, China) and carried out code optimization (Additional file 1). Then, we digested these constructs with XbaI (Takara, Japan) and HindIII (Takara, Japan), cloned the p54 gene and p54-pIL-21 fusion gene into the pSIP-409-pgsA' vector, and transformed the positive plasmid into *L. plantarum* strain NC8 (CCUG 61730). Two recombinant *L. plantarum* strains, NC8-pSIP409-pgsA'-p54 and NC8-pSIP409-pgsA'-p54-pIL-21, were obtained. They were sequenced and identified by Shanghai Shenggong Biotechnology Co., Ltd. (Shanghai, China).

### Preparation of anti-p54 antibody

The p54 gene sequence was ligated into the pET28a expression vector and transformed into BL21 to obtain recombinant *Escherichia coli* BL21-pET-28a-p54. p54 protein expression was induced by IPTG (100 mM) (Sigma, Japan). The p54 protein was purified and recovered. Rabbit polyclonal antibodies were obtained by immunizing rabbits with purified p54 protein and used to detect the expression of bacterial target genes.

### Western blotting

To detect the expression of p54 and p54-pIL-21 antigens in *L. plantarum* NC8, NC8-pSIP409-pgsA'-p54 and NC8-pSIP409-pgsA'-p54-pIL-21 were cultured with 10 μg/mL erythromycin and 50 ng/mL sakacin P inducer (SppIP) at 30 °C for 9 h. After separation by SDS-PAGE (10% acrylamide), the bacterial protein was transferred to a nitrocellulose membrane and incubated with the polyclonal rabbit anti-p54 antibody and then a goat anti-rabbit secondary antibody coupled with horseradish peroxidase (HRP) (CST, USA). After washing, protein detection was visualized by enhanced chemiluminescence (ECL, USA) on an Amersham Imager (General Electric Company).

### Flow cytometry assay and immunofluorescence detection

The recombinant strain *L. plantarum* (0.5 mL) induced by the above method was resuspended in PBS and diluted to the appropriate OD value, incubated with the rabbit anti-p54 antibody(BD Bioscience, U.S.A) at 4 °C for 1 h, and then stained with a goat anti-mouse IgG labeled with FITC at 4 °C for 1.5 h. After washing with PBST, the recombinant bacterial cells were evaluated by flow cytometry (BDLSR Fortessa cells (USA) and fluorescence microscopy (Leica DMI8, Germany)).

### Immunization

To evaluate the immune effect of recombinant *L. plantarum* NC8-pSIP409-pgsA'-p54 and NC8-pSIP409-pgsA'-p54-pIL-21, 40 C57BL/6 mice were divided into four groups. The mouse group was immunized with 200 μL with colony-forming units (1 × 10^9^ CFU) of NC8-pSIP409-pgsA', NC8-pSIP409-pgsA'-p54 or NC8-pSIP409-pgsA'-p54-pIL-21 suspension by oral gavage. The control group was given 0.9% normal saline (200 μL) by the same method. The mice were first immunized on the 1st, 2nd and 3rd days, then received booster immunization on the 14th, 15th and 16th days, and finally boosted again on the 28th, 29th and 30th days. The feces and serum of each mouse were collected for ELISA detection on the 13th, 27th and 43rd days.

### Flow cytometry

Nine mice in each group were euthanized on the 14th day after the last immunization. Single-cell suspensions of spleen (SP), mesenteric lymph nodes (MLNs) and Peyer's patches (PPs) of all groups of mice were prepared as previously described (Huang et al. 2018). After preparing single-cell suspensions from MLNs and PPs, we transferred 10 μL of anti-B220 antibody (BD Bioscience, USA) into a test tube containing 1 × 10^6^ cells, mixed the solution well and stained the cells in the dark at 4 °C for 30 min. We added 1 mL of phosphate-buffered saline (PBS) to the collected samples. The cell suspension was centrifuged at 2000 rpm and 4 °C for 5 min, and the supernatant was discarded. The above steps were repeated. The cells were then fixed and permeabilized, centrifuged twice, and stained for 30 min with 10 μL of anti-IgA antibody (BD Bioscience, USA) at 4 °C in the dark using the same procedure described above. Single-cell suspensions of MLNs and SPL were transferred to a 48-well cell culture plate, and the cells were incubated with the p54 protein for 8 h. Then, inhibitors were added for 3 h. The cells were centrifuged twice, and 10 μL of anti-CD3, anti-CD4 and anti-CD8 antibodies (BD Biosciences, USA) were added. The cells were then fixed, permeabilized, centrifuged twice, and mixed with 10 μL anti-IFN-γ antibody (BD Biosciences, USA) for 30 min at 4 °C in the dark. BD fluorescence-activated cells were sorted and analyzed by FACS in an LSRFortessa analyzer (BD Bioscience, USA). All data were analyzed using FlowJo 7.6 software.

### Enzyme-linked immunosorbent assay

The presence of antigens that specifically bound to IgG and IgA antibodies in serum and Fecal supernatant was assessed by Enzyme-linked immunosorbent assay (ELISA), with some minor alterations (Shi et al. 2014). Briefly, 96-well polystyrene microtiter plates were coated with 1 μg p54 antigen using carbonate-bicarbonate buffer (pH9.6) and incubated overnight at 4 °C. Then sealed with 150 μL of blocking solution (PBST containing 10% bovine serum albumin) at 37 °C for 1 h. The wells were washed three times with PBST. The diluted samples were added to the well, incubated at 37 °C for 2 h, and then washed three times with PBST. 100 μL of anti-mouse-HRP conjugate (CST, USA) was added to all the wells at a dilution of 1:5000 and incubated for 1 h. After washing four times with wash buffer, the plate was developed with 0.02% *O*-phenylenediamine and 0.015% H_2_O_2_ (Zymed) in substrate buffer (15 mM citrate buffer pH 5.6), and the reaction was stopped after 10 min with 2N H2SO4. The absorbance was read at 492 nm. Finally, termination solution is added to terminate the reaction. The OD value was determined by an enzyme labeling instrument. The final titer was evaluated as the highest dilution, resulting in twice the absorbance of the sample background.

### Lymphocyte proliferation test

To evaluate the proliferation of primary immunized lymphocytes, MLN cells (2 × 10^5^ cells) and SLP cells (2 × 10^5^ cells) were placed in 96-well plates, and p54 antigen (5 µg/mL) was added to each well. After 72 h, the cells were removed, and 20 μL of MTS was added to each well to stop the reaction. After 4 h, the OD value was detected by an enzyme labeling instrument.

### Statistical analysis

All the data in the experiment came from at least three independent experiments and are expressed as the average ± SEM. GraphPad Prism 5.0 software was used to test the differences. P < 0.05 was considered to represent a significant difference. Analysis of variance (ANOVA) with Tukey's multiple comparison test was used to evaluate the significance.

## Results

### Construction of plasmids and expression of target genes in vitro

**Fig. 1:**
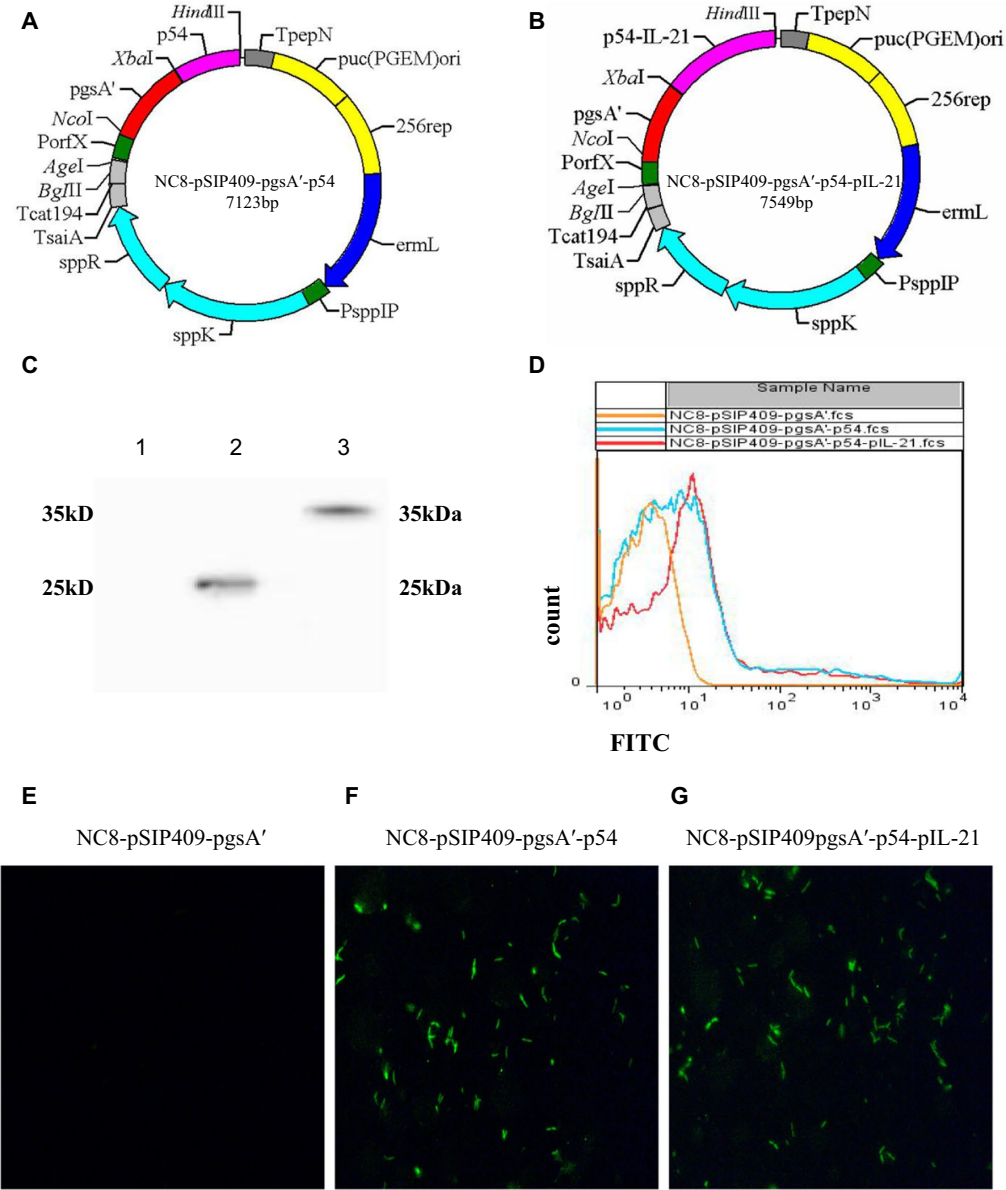
**A** A map of the plasmid NC8-pSIP409-pgsA'-p54; **B** a map of the plasmid NC8-pSIP409-pgsA'-p54-pIL-21; **C** the expression of the recombinant *L. plantarum* ASFV p54 protein and p54-pIL-21 fusion protein was verified by Western blot, lane 1: NC8-pSIP409-pgsA', lane 2: NC8-pSIP409-pgsA'-p54, lane 3: NC8-pSIP409-pgsA'-p54-pIL-21; **D** the expression of recombinant *Lactobacillus plantarum* was verified by immunofluorescence; **E** the anchored expression of the ASFV p54 protein and p54-pIL-21 fusion protein on the surface of *Lactobacillus plantarum* was verified by immunofluorescence

Two recombinant *L. plantarum* NC8-pSIP409-pgsA'-p54 and NC8-pSIP409-pgsA'-p54-pIL-21 strains (Fig. 1A) were successfully constructed. Rabbit polyclonal antibodies were used to bind to the target protein p54 and p54-pIL-21 fusion protein and then incubated with a goat anti-rabbit secondary antibody (HRP labelled) (Fig. 1B). The WB results showed that the target band was consistent with the expected size, which proved that the rabbit-specific antibody was obtained by immunizing rabbits with purified p54 antigen and that the recombinant *L. plantarum* successfully expressed foreign protein (Fig. 1C). The results of immunofluorescence detection showed that the FITC-labeled secondary antibody could combine with the rabbit primary antibody; thus, the surface-anchored expression of the p54 protein and p54-pIL-21 fusion protein of *L. plantarum* was ideal, and the expression efficiency was high (Fig. 1D). The protein expression of *L. plantarum* was detected by flow cytometry after induction. The results showed the anchored expression (Fig. 1E) of *L. plantarum* on the surface of the bacteria.

### Mucosal immunity induced by recombinant *L. plantarum*

**Fig. 2:**
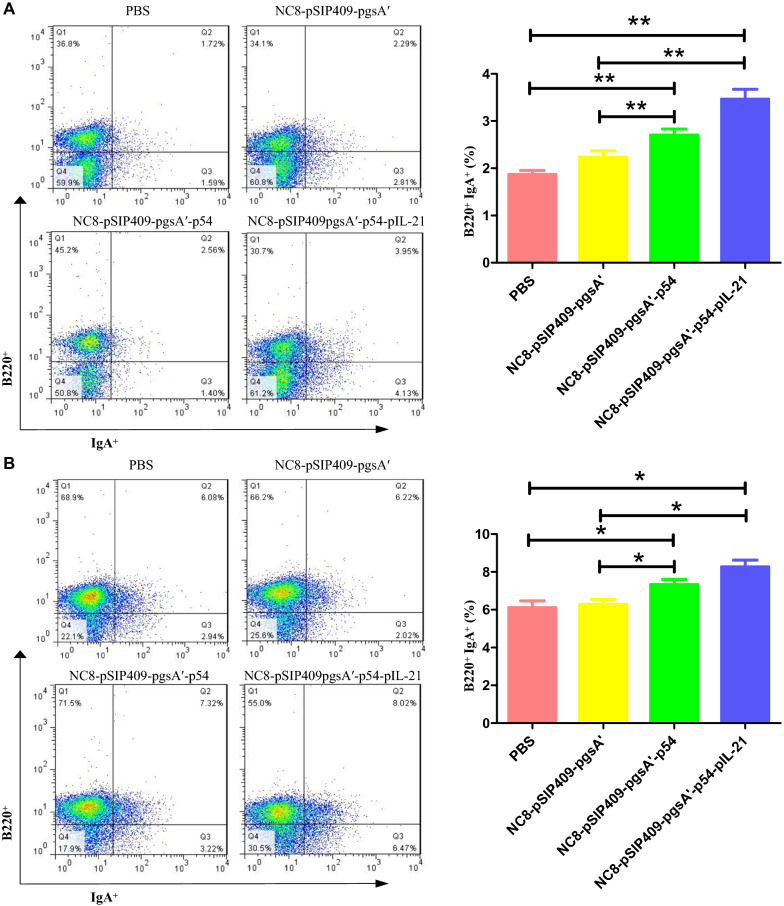
**A** Statistical analysis of lymphocyte expression of B220 and IgA in mouse PPs; **B** statistical analysis of lymphocyte expression of B220 and IgA in mouse MLNs

**Fig. 3:**
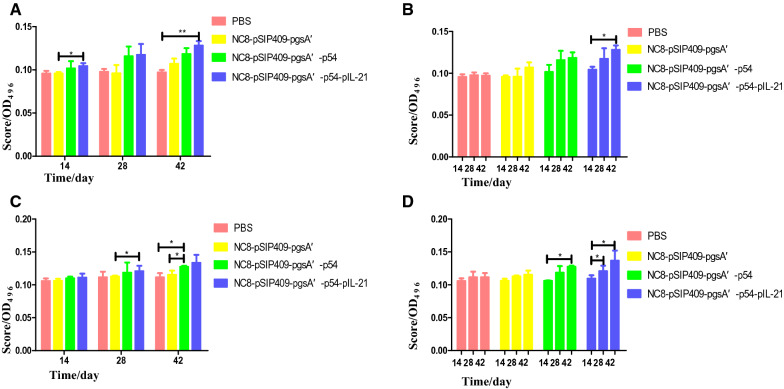
**A** Comparison of the increase in fecal IgA content in the four groups of mice after three immunizations. **B** Comparison of the increase in the fecal IgA content of the four groups of mice after each immunization. **C** Comparison of the increase in the fecal IgG content of the four groups of mice after three immunizations. **D** The increase in the fecal IgG content of the four groups of mice after each immunization

Recombinant *L. plantarum* significantly enhanced the detection of B220^+^ IgA^+^ cells in PPs (Fig. 2A) and MLNs (Fig. 2B). The results of IgA detection in the feces of mice showed that after the first immunization, the IgA content in the feces of the mice fed NC8-pSIP409-pgsA'-p54-pIL-21 was significantly higher than that of the control mice. After the third immunization, the IgA content in the feces of mice fed NC8-pSIP409-p54-IL-21 was significantly higher than that of mice fed PBS (Fig. 3A). The results of IgA detection in the feces of mice showed that there was no significant change in the mice fed PBS after three immunizations; the IgA content in the feces of the mice fed NC8-pSIP409-pgsA' and the mice fed NC8-pSIP409-pgsA'-p54 increased slightly, but the difference was not significant; the IgA content in the feces of mice fed NC8-pSIP409-pgsA'-p54-pIL-21 increased significantly (Fig. 3B).

### Increase in IgG content in serum induced by recombinant *L. plantarum*

The results of serum IgG detection showed that there was no significant difference in serum IgG among the four groups after the first immunization. The IgG content in the serum of mice fed NC8-pSIP409-pgsA'-p54 and NC8-pSIP409-pgsA'-p54-pIL-21 was slightly higher than that of the control and blank mice, but the difference was not significant. After the second immunization, the IgG content in the serum of mice fed NC8-pSIP409-p54-pIL-21 was significantly higher than that of the control mice. After the third immunization, the IgG content in the serum of mice fed NC8-pSIP409-pgsA'-p54 was significantly higher than that of mice fed PBS or NC8-pSIP409 (Fig. 3C). The results of serum IgG detection showed that there was no significant change in the serum IgG content of mice fed PBS after three immunizations, but the IgG content in the serum of mice fed the NC8-pSIP409-pgsA' group increased slightly, but the difference was not significant, while the IgG content in the serum of mice fed NC8-pSIP409-pgsA'-p54 and mice fed NC8-pSIP409-pgsA'-p54-pIL-21 increased significantly (Fig. 3D).

### Cellular immune response induced by recombinant *L. plantarum*

**Fig. 4:**
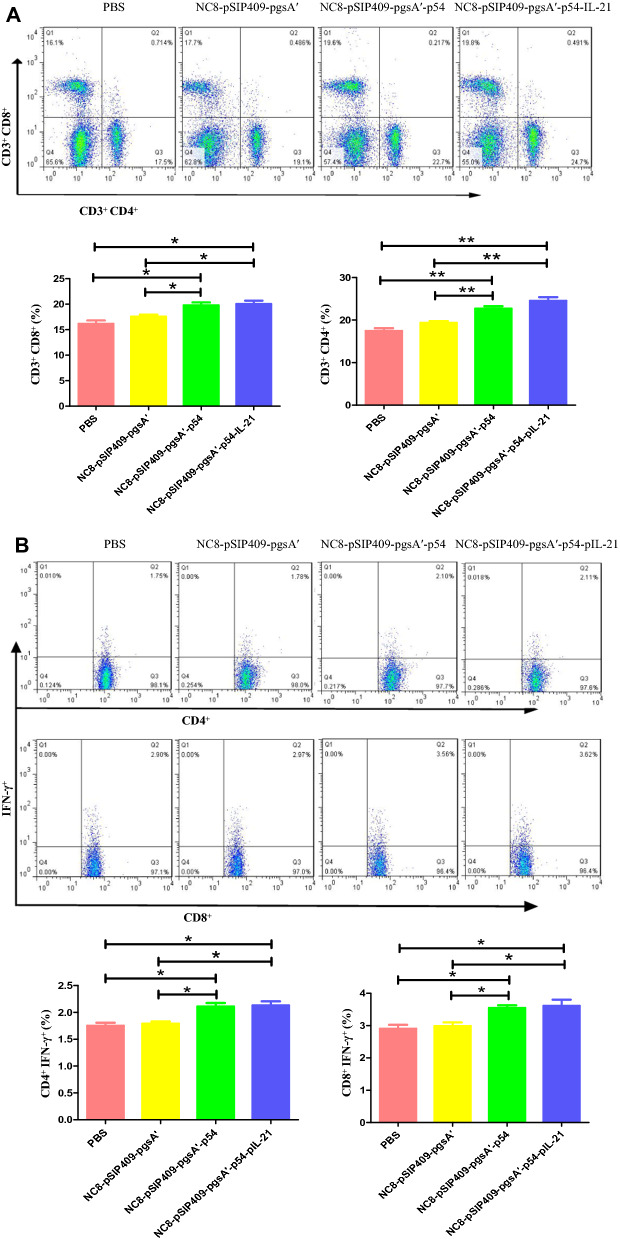
**A** Changes in the detection of CD3^+^ CD4^+^ and CD3^+^ CD4^+^ T cells in the SP; **B** changes in CD4^+^ IFN-γ^+^ and CD8^+^ IFN-γ^+^ T cells in the MLN

**Fig. 5:**
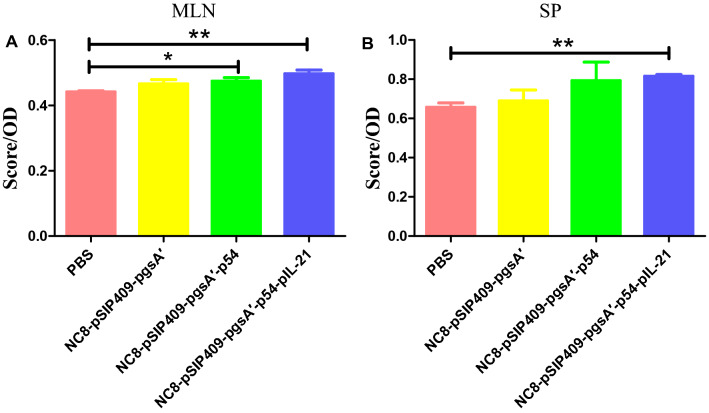
**A** The results of lymphocyte proliferation in the MLN. **B** The results of lymphocyte proliferation in the SP

Recombinant *L. plantarum* enhanced immunity, and the contents of CD3^+^ CD4^+^ and CD3^+^ CD8^+^ T cells in SP lymphocytes increased significantly (Fig. 4A). The MLN is one of the main mucosa-associated lymphoid tissues, in which the level of activated lymphocytes is an important marker for evaluating immunity. IFN-γ can be produced when T cells are stimulated by antigens, which can widely neutralize viruses and play an important role in immune regulation. Recombinant *L. plantarum* enhanced the detection of CD4^+^ IFN-γ^+^ and CD8^+^ IFN-γ^+^ T cells in the MLN (Fig. 4B). The results of MLN lymphocyte proliferation showed that the mice fed recombinant *L. plantarum* NC8-pSIP409-pgsA'-p54 and NC8-pSIP409-pgsA'-p54-pIL-21 had significantly greater proliferation than those fed PBS (Fig. 5A). The results of SP lymphocyte proliferation showed that the mice fed recombinant *L. plantarum* NC8-pSIP409-pgsA'-p54-pIL-21 had significantly greater proliferation than those fed PBS (Fig. 5B). There are a certain number of memory lymphocytes in mice stimulated by p54 antigen and p54-pIL-21 fusion antigen that can proliferate and produce specific antibodies to bind to them when recognizing antigens.

## Discussion

ASF is a complex pig disease that is poorly controlled in most affected areas due to a lack of vaccines. Therefore, it is urgent to develop a reasonably protective ASF vaccine (Arias et al. 2017). It is known that all kinds of ASFV vaccines have not achieved good results, but some scholars have proposed the advantages and feasibility of subunit vaccines (Gaudreault and Richt 2019). At the same time, this experiment used *L. plantarum* to express the ASFV P54 antigen in mice and achieved a good immune effect.

Vaccination through the mucosal route is helpful to target the induced site of the SIgA response and greatly enhance the mucosal immune response, which is the most effective way to trigger a protective mucosal immune response (Boyaka. 2017; Jin et al. 2019). For example, a human mucosal antiinfluenza vaccine can induce high local humoral and cellular immune responses (Calzas and Chevalier 2019); a mucosal vaccine constructed with the HIV envelope protein induces HIV-specific immunity in the blood and mucosa where the virus enters (Chege et al. 2017). Studies have shown that microbial antigens can induce antigen-specific mucosal and systemic immune responses in mice and can be used to prevent microbial infections in the intestinal tract (Fujimoto et al. 2019). The *L. plantarum* strain used in this study can protect the intestinal barrier and activate the proliferation of intestinal epithelial cells (Hou et al. 2018). *L. plantarum* has good application prospects as a mucosal vaccine carrier. In our experimental results, we found that recombinant *L. plantarum* can significantly increase the secretion of SIgA from the intestinal mucosa. In the past decade, the successful development of mucosal vaccines against influenza virus and rotavirus infection has increased people's interest in this field, and people have high hopes for new mucosal vaccines (Lycke 2012).

It is well known that the increase in IgG content in body fluid helps to improve the antiviral activity of the body. Specific IgG was detected from 15 to 21 days after pigs were immunized with African classical swine fever live attenuated vaccine and maintained at a high level during the experiment (Sánchez-Cordón et al. 2018). An adenovirus vector vaccine containing the ASFV antigen can induce an increase in IgG content in the body fluid of pigs and has good immunogenicity (Lokhandwala et al. 2019). In our study, the IgG content in the blood of mice in the experimental group increased significantly after each immunization and reached the highest value 14 days after the third immunization, which was consistent with the humoral immunity induced by the subunit vaccine expressing foot-and-mouth disease in Holstein cattle (Sitt et al. 2019). Some researchers immunized mice with the ASFV structural protein p72 recombinant Newcastle disease virus vaccine to produce a high-titer specific IgG antibody (Chen et al. 2016). Therefore, the increase in IgG content in body fluid contributes to the increase in anti-ASFV activity.

ASFV infection of animal lymphocytes results in a decrease in lymphocyte activity and can negatively regulate the expression of interferon, which plays an important role in viral immune escape (Wang et al. 2018a, b). The proliferation of CD4^+^ and CD8^+^ T cells is affected (Childerstone et al. 1998). It is known that helper (CD4^+^) T cells play an important role in inducing the mucosal antibody response (Aljurayyan et al. 2018). In addition, studies have shown that CD8^+^ lymphocyte subsets play an important role in ASFV protective immunity (Takamatsu et al. 2013). Protective immunity against African classical swine fever virus is lost when CD8^+^ T lymphocytes are depleted in the body (Oura et al. 2005). Some studies have shown that interferon-γ can inhibit the replication of African classical swine fever virus (Fan et al. 2020), and T cells proliferate and increase the secretion of interferon-γ in mice immunized with ASFV structural protein (Chen et al. 2016). Interestingly, in our study, we also found that the number of activated CD4^+^ T cells and CD8^+^ T cells in the experimental group increased, the secretion of IFN-γ in CD4^+^ and CD8^+^ T cells increased, and lymphocytes proliferated under stimulation with the ASFV p54 antigen. Other studies have shown that vaccines that induce a high-intensity CD8^+^ T cell response may enhance the protective effect against HIV virus (Petitdemange et al. 2019).

Interleukin-21 (IL-21) plays an important role in both innate and acquired immune responses, and its expression is significantly increased in a variety of viral infections. The specific receptor IL-21R is produced and localized on the surface of T and B cells and natural killer (NK) cells, which plays a key role in the proliferation and differentiation of these immune effector cells (Shoraka et al. 2019). IL-21 can also increase the number of helper (CD4^+^) T cells and germinal center B cells, thus increasing the immunity of the body (Zhang et al. 2016). Some studies have shown that exogenous methods to increase IL-21 production or increase cellular IL-21 production can limit the degree of initial HIV-1 infection (Ortega et al. 2018). A number of studies have shown that IL-21 plays an important role in antiviral humoral immunity (Rasheed et al. 2013). Because of the high homology between porcine IL-21 and mouse IL-21 (approximately 80%), the immune effect of mice fed recombinant *L. plantarum* expressing p54 antigen and pIL-21 in our experiment is also the most significant, and related experiments will be carried out in pigs in the future.

In this study, we produced recombinant *L. plantarum* expressing a ASFV p54 and p54-pIL-21 fusion protein and evaluated the immune effect of NC8-pSIP409-pgsA'-p54 and NC8-pSIP409-pgsA'-p54-pIL-21 in a mouse model. The results showed that recombinant *L. plantarum* could improve the humoral immunity, cellular immunity and mucosal immunity of mice, which laid a theoretical foundation for the development of oral vaccines against ASFV. For the phenomenon of ASFV infection through the mucosa, the mucosal vaccine may have a certain effect on blocking the virus and provide guidance for our further experiments in pigs.

## Supplementary Information

**Additional file 1.** The optimized sequence of p54.

## Data Availability

The raw data reported in the present paper have been deposited in Jilin Agricultural University, Chang Chun, China.
