# Supplementary material for: Lactobacillus plantarum surface-displayed ASFV (p54) with porcine IL-21 generally stimulates protective immune responses in mice
Source: AMB Express. 2021 Aug 12;11:114. doi: 10.1186/s13568-021-01275-9 (PMC8360262; doi:10.1186/s13568-021-01275-9)
Supplement: Supplementary file 1 — Additional file 1. The optimized sequence of p54. [file 13568_2021_1275_MOESM1_ESM.docx]

The Optimized sequence of p54

A 133 T 100 C 163 G 156 | GC%: 57.79% | Length: 552

ATGGATAGCGAATTTTTTCAGCCGGTGTATCCGCGCCATTATGGCGAATGCCTGAGCCCGGTGACCCCGCCGAGCTTTTTTAGCACCCATATGTATACCATTCTGATTGCGATTGTGGTGCTGGTGATTATCATTATTGTGCTGATTTATCTGTTTAGCAGCCGCAAAAAGAAAGCGGCCGCGGCCATTGAAGAGGAAGATATTCAGTTTATTAACCCGTATCAAGATCAGCAGTGGGCCGAAGTGACCCCGCAACCGGGCACGAGCAAACCGGCGGGCGCGACCACCGCGAGCGCGGGTAAACCGGTTACCGGTCGCCCGGCGACCAACCGTCCGGCGACGAACAAACCGGTTACCGACAACCCGGTTACCGATCGCCTGGTGATGGCGACCGGCGGCCCGGCGGCGGCGCCGGCGGCGGCGAGCGCGCATCCGACCGAACCGTATACCACCGTGACCACGCAGAACACCGCGAGTCAGACCATGAGCGCGATTGAAAACCTGCGTCAGCGCAACACCTATACCCATAAAGATCTGGAAAACAGCCTGTAA

**GC Content**


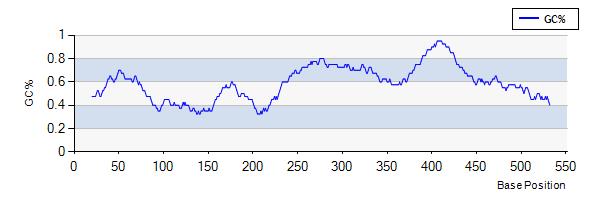


**Direct Repeat**

| Len | Sequence | Sites |  |
| --- | --- | --- | --- |
| 13 | AAACCGGTTACCG | 301, 346 |  |
| 12 | CCGGCGGCGGCG | 400, 412 |  |

**Short Dyads**

| Len | Sequence | Sites |  |
| --- | --- | --- | --- |
| 15 | CGGCGGCCCGGCGGC | 393 |  |
| 13 | CCATATGTATACC | 87 |  |
